# Control of the surface plasmon dispersion and Purcell effect at the metamaterial-dielectric interface

**DOI:** 10.1038/s41598-020-77688-6

**Published:** 2020-11-30

**Authors:** Konstantin A. Ivanov, Konstantin M. Morozov, Galia Pozina, Azat R. Gubaydullin, Elizaveta I. Girshova, Mikhail A. Kaliteevski

**Affiliations:** 1 ITMO University, St. Petersburg, Russia 197101; 2 St. Petersburg Academic University, St. Petersburg, Russia 194021; 3 Department of Physics, Chemistry and Biology (IFM), Linköping University, 58183 Linköping, Sweden; 4 Department of Applied Physics, Aalto University School of Science, P.O. Box 13500, 00076 Aalto, Finland

**Keywords:** Metamaterials, Photonic crystals, Nanophotonics and plasmonics

## Abstract

The use of metamaterial as a way to mitigate the negative effects of absorption in metals on the Purcell effect in metal-dielectric structures is investigated. A layered metal-dielectric structure is considered as an anisotropic medium in the long-wavelength limit. The dispersion of the surface plasmon appearing at the boundary between such a structure and a different dielectric material, as well as the position of the peak in the local density of states are studied for various combinations of materials and filling factors of the periodic structure. The calculated frequency dependence of the Purcell factor demonstrates an increase in peak value compared to the conventional plasmonic structure. The results obtained using effective media approach are compared to the results of numerical modelling.

## Introduction

Surface plasmon, a localized state of an electromagnetic field at the interface between a metal and a dielectric, was predicted more than sixty years ago^1^. Due to the formation of such states, metal-dielectric structures can facilitate a strong light-matter interaction and therefore attract significant research interest^2–5^. For example, field localization provides possibilities for the development of subwavelength optical devices^6,7^, while the increased amplitude of the field near the interface allows to utilize such systems in sensor devices^8,9^. Moreover, a number of spectacular effects caused by surface plasmons can be mentioned^10–13^, in particular, a surface-enhanced Raman scattering^14,15^ and the Purcell effect^16^, which is the enhancement of spontaneous emission probability in an inhomogeneous medium. The latter phenomenon is crucial for increasing the efficiency of light emission in optoelectronic and photonic devices^17,18^.

Previously, it was proposed that the major enhancement of the spontaneous emission probability can be achieved in plasmonic structures due to a high local density of states (LDOS)^19^. However, this conclusion has been argued^20^ in recently published paper^21^, where it was demonstrated that for the frequency range, where the LDOS peak is occurring, this enhancement is dramatically reduced due to light absorption in metals. Nevertheless, the existence of features in the dispersion curve suggests that the effective utilization of the surface plasmons still can be achieved^22^.

A possible way to obtain higher magnitudes of the enhancement of the spontaneous emission probability is to shift the LDOS peak to the low frequency range, where absorption is lower via application of “effective plasma frequency” (EPF) concept^23,24^. In such materials bulk metal is replaced with structured one and EPF defines the properties of the structures instead of plasma frequency of metal. Shifting of dispersion dependence towards lower energy is demonstrated for various 2D and 3D plasmonic metamaterials (MM)^25–28^, and for layered metamaterials^29–31^. It was also shown theoretically and experimentally that in layered metamaterials photonic LDOS can be enhanced^32–38^ leading to a pronounced Purcell effect. These works deal with various aspects of the Purcell effect in metamaterials but are similar since they are always considered the dipole placed at some distance from the interface, which is either arbitrary or dictated by experimental or numerical conditions. Further, the dependence of the value of the Purcell factor on the metal filling factor seems to be out of scope of aforementioned papers creating a gap between dispersion studies^25–31^ and Purcell effect studies, which we aim to cover. Also, it is interesting to investigate limits of applicability of effective media approach to the analysis of the Purcell effect in metamaterial-based structures.

This paper is aimed at the investigation of the parameters of the metamaterials and dielectric on the spectrum of the Purcell coefficient using effective media approach and numerical modelling.

### Results and discussion

**Figure 1:**
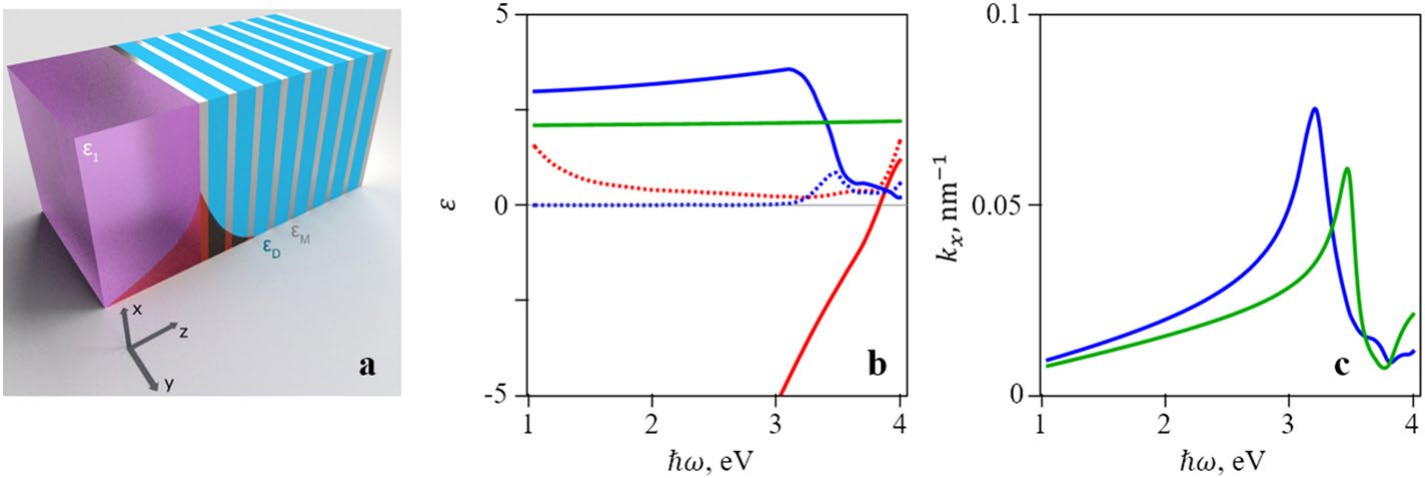
(**a**) Geometry and composition of the structure. Silver and blue colors denote metallic and dielectric layers of metamaterial, respectively. The purple color denotes dielectric cladding. The red curve represents the electric field of the surface plasmon. (**b**) Dielectric constant dispersion in materials used in calculations (solid—real part, dashed—imaginary part): red—silver, blue—CBP, green—silica. (**c**) dispersion of the surface plasmon on the interface silica/silver (green) and CBP/silver (blue). (**a**) was created in Blender (ver. 2.69, https://www.blender.org/). Figures (**b**) and (**c**) were created in Wolfram Mathematica (ver. 12, https://www.wolfram.com/). Images were combined in Microsoft PowerPoint 365 (https://www.office.com/).

The proposed metamaterial is a one-dimensional structure consisting of alternating layers of metal and dielectric parallel to the $xy$ plane (see Fig. 1a). The dielectric functions are labeled by $\varepsilon_{Me}$. and $\varepsilon_{D}$, and the corresponding layer thicknesses by $a$ and $b$, respectively. We denote the fraction of metal in the metamaterial, i.e. the filling factor, as

1 $$\alpha = \frac{a}{a + b}$$

It is known that in the long-wavelength limit, which is satisfied for the low-frequency range, such a structure can be considered as a uniform anisotropic medium with a dielectric tensor:

2 $$\left( {\begin{array}{*{20}c} {\varepsilon_{x} } & 0 & 0 \\ 0 & {\varepsilon_{x} } & 0 \\ 0 & 0 & {\varepsilon_{z} } \\ \end{array} } \right)$$

Note that only two different non-zero components are present. They are defined as:

3a $$\varepsilon_{x} = \alpha \varepsilon_{M} + \left( {1 - \alpha } \right)\varepsilon_{D} ,$$

3b $$\frac{1}{{\varepsilon_{z} }} = \frac{\alpha }{{\varepsilon_{M} }} + \frac{1 - \alpha }{{\varepsilon_{D} }}$$

In such medium, two possible types of waves can propagate: ordinary and extraordinary. If this metamaterial is stacked with a cladding with dielectric constant $\varepsilon_{1}$ (see Fig. 1a), two surface plasmon states will appear, which we will accordingly call ordinary and extraordinary plasmons.

**Figure 2:**
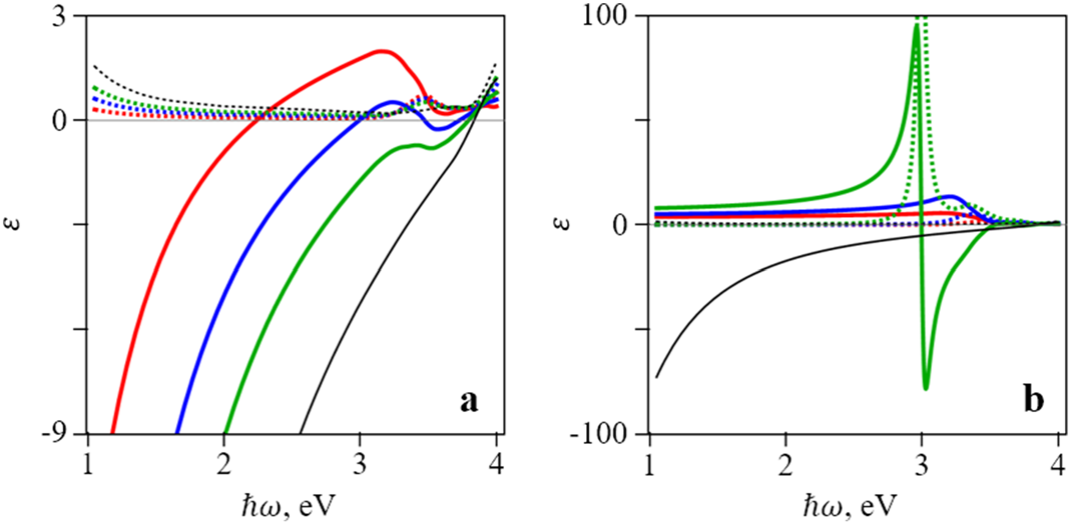
Dependence of real (solid lines) and imaginary (dashed lines) parts of the dielectric tensor components: (**a**) $\varepsilon_{x}$ and (**b**) $\varepsilon_{z}$ on frequency for different values of $\alpha$ (red: $\alpha = 0.2$, blue: $\alpha = 0.4$, green: $\alpha = 0.6$) in a silver/CBP metamaterial. Black lines denote dispersion of silver dielectric constant. Plots were created in Wolfram Mathematica (ver. 12, https://www.wolfram.com/) and combined in Microsoft PowerPoint 365 (https://www.office.com/).

In the following, we demonstrate how the proposed structure can be used to control the properties of the metamaterial, the dispersion of a surface plasmon, and the value of the Purcell factor. We will use silver as an example of metal. The value of its dielectric constant is taken from the experimental work^39^. As for dielectric, we will use several materials. One of them is the organic light-emitting compound 4,4-bis(*N*-carbazolyl)-1,1-biphenyl (CBP) that was previously studied for its suitability for silver-based plasmonic structures^40^: CBP has a wide emission band in the photon energy interval 2.7 eV to 3.5 eV. CBP is used as an example of a filling material and silica as a cladding material. Dispersion of the real and imaginary parts of dielectric constants of silver, silica and CBP are shown in Fig. 1b. Note, that real part of dielectric constant of silver becomes equal to zero for photon energy 3.82 eV. Figure 1c shows the dispersion of surface plasmons on the interface of silver/silica and silver/CBP. It can be seen that for silver/silica (silver/CBP) interface the photon energy of the feature, corresponding to surface plasmon is 3.47 eV (3.2 eV). A straightforward calculation of the dispersion of dielectric tensor components using Eq. (3) gives the results shown in Fig. 2. An important feature of the $\varepsilon_{z}$ dependence is a double peak and a change of sign at a sufficiently large value of $\alpha$.

**Figure 3:**
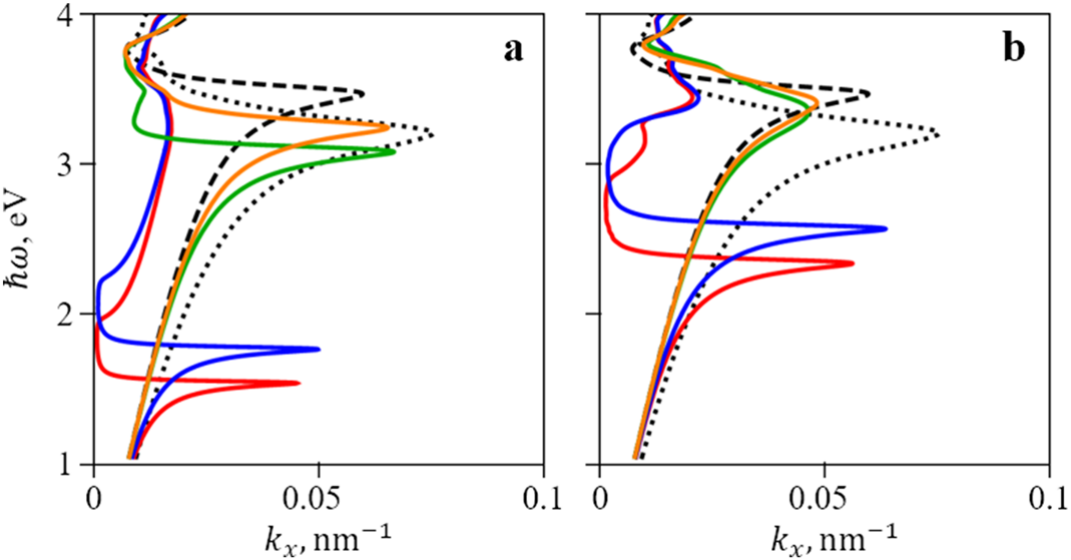
Dispersion of the surface plasmon at the interface between silver/CBP metamaterial and silica: ordinary surface plasmon (**a**), extraordinary surface plasmon (**b**). Colors denote different values of $\alpha$ (red: $\alpha = 0.15$, blue: $\alpha = 0.2$, green: $\alpha = 0.7$, orange: $\alpha = 0.8$) in a silver/CBP metamaterial. Black lines denote dispersion of a simple plasmon at the interface between silver and CBP (dotted line) and silica (dashed line). Plots were created in Wolfram Mathematica (ver. 12, https://www.wolfram.com/) and combined in Microsoft PowerPoint 365 (https://www.office.com/).

As we analytically show in the “Methods” section in Eqs. (11) and (12), reducing the fraction of metal in the metamaterial leads to shift of the LDOS peak frequency towards lower frequencies. The reason for that is that lowering the fraction of metal effectively reduces the plasma frequency, which determines the LDOS peak position. The surface plasmon dispersion calculated according to Eqs. (6) and (8) presented in “Methods” is shown in Fig. 3. For comparison, the dispersion of a simple surface plasmon at the interface between silver and both used dielectrics (CBP and silica) is also shown. Importantly, the plasmon associated with an ordinary wave is proved to be more effective in reducing the peak frequency of LDOS. This is because the ordinary wave is essentially propagating in a material with $\varepsilon = \varepsilon_{x}$, which is equivalent to a metal with reduced plasma frequency. Extraordinary wave mixes $\varepsilon_{x}$ with $\varepsilon_{z}$, which, as can be seen in Fig. 2, is similar to that of a dielectric. This reduces the effect of the lowered plasma frequency. It is obvious that by varying the filling factor $\alpha$ one can tune the peak frequency to any wavelength.

**Figure 4:**
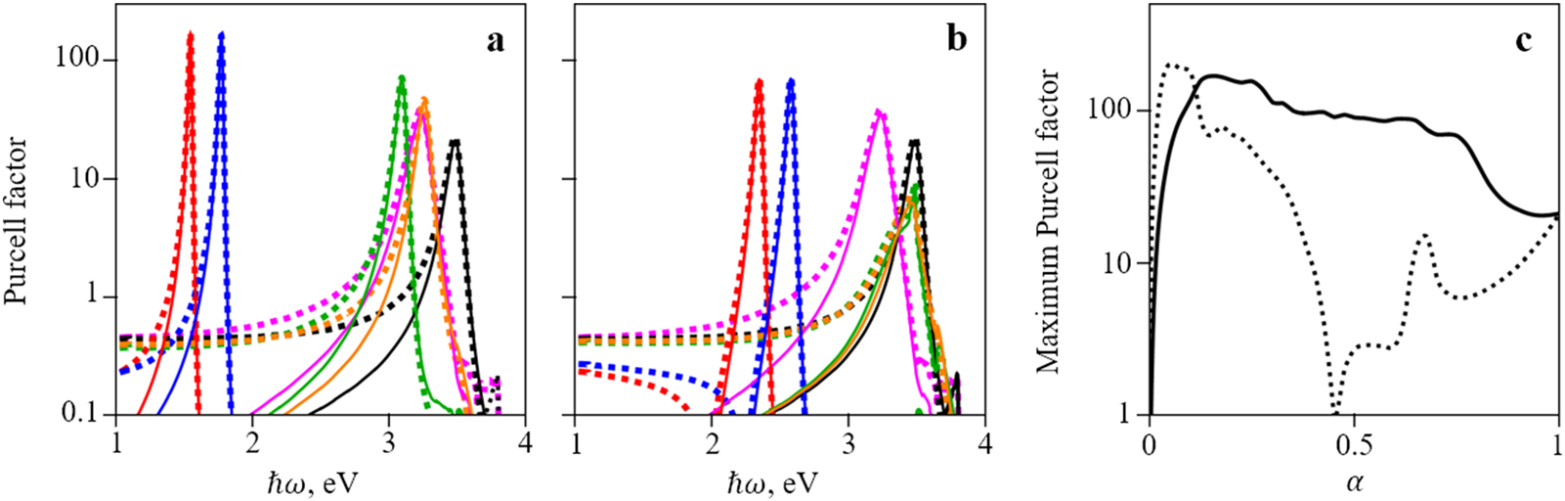
(**a**,**b**) Dependence of the Purcell factor on frequency for an interface between silver/CBP metamaterial and silica for the ordinary surface plasmon (**a**) and the extraordinary surface plasmon (**b**). Solid lines represent orientation of the dipole parallel to the layers, dotted lines—perpendicular to the layers. Colors denote different values of $\alpha$ (red: $\alpha = 0.15$, blue: $\alpha = 0.2$, green: $\alpha = 0.7$, orange: $\alpha = 0.8$) in a silver/CBP metamaterial. Black and magenta lines denote Purcell factor of a simple plasmon for an interface between silver and CBP (magenta line) and silica (black line). (**c**) Dependence of the maximum Purcell factor on the value of $\alpha$ for the same structure for dipole parallel to the layers. Ordinary surface plasmon (solid lines), extraordinary surface plasmon (dotted lines). Plots were created in Wolfram Mathematica (ver. 12, https://www.wolfram.com/) and combined in Microsoft PowerPoint 365 (https://www.office.com/).

The results of the Purcell factor calculation are shown in Fig. 4a,b, where, for comparison, the values of the Purcell factor for a simple interface plasmon are also shown, and in Fig. 4c where the maximum value of the Purcell factor is plotted against the filling factor $\alpha$. Clearly, lowering the LDOS peak frequency towards the low-absorption range is proven to be effective. The value of the Purcell factor can be easily increased tenfold. Importantly, a decrease in the value of the filling factor $\alpha$ increases the value of the Purcell factor for an ordinary plasmon. For very small values of $\alpha$ the maximum Purcell factor is approaching zero as there can be no surface plasmon on a dielectric-dielectric boundary (since at $\alpha = 0$ the metamaterial is nothing more than a dielectric). When $\alpha$ is increasing in the middle of its range, the value of the Purcell factor tends to decrease due to higher absorption and penetration of the electric field into the metamaterial half of the structure. Finally, at $\alpha = 1$ the maximum value of the Purcell factor is equal to the value for the conventional metal–dielectric interface plasmon as expected. Between the ordinary- and extraordinary-wave plasmons, the former is again more effective in enhancing the Purcell effect. This is again the consequence of the fact that the extraordinary wave “mixes” $\varepsilon_{x}$ (which is metal-like) with $\varepsilon_{z}$ (dielectric-like). Since this reduces the efficiency of the LDOS peak lowering, this also means that the LDOS peak resides in a frequency area where the metal absorbs light better. This lowers the value of the Purcell factor. The complex shapes of the curves in Fig. 4c are a consequence of the irregularities in the refractive index values for silver and CBP, to which the peak Purcell factor value is very sensitive. There is almost no difference in peak Purcell factor value for different orientations of the dipole (parallel and perpendicular to the interface), contrary to the results reported by Kala et al.^35^, where dipole orientation along interface provides negligible Purcell factor values. This discrepancy is, however, the result of different geometries of structures considered in our work and in^35^. In^35^ the structure was symmetrical, and the dipole was placed in its center; as a result, the x-component of the electric field vanishes obliterating Purcell effect. Here, we consider different geometry, in which both components have almost the same value due to the lack of symmetry.

It is important to note that plotted value corresponds to the dipole being placed directly on the interface. As the plasmonic state’s field decreases exponentially when moving away from the interface, the value of the Purcell factor will inevitably be lower if the dipole has some misplacement. In this work, however, we only investigate the maximum Purcell factor.

Analysis of the Purcell coefficients at the interface between MM and dielectric using effective media approach presented above shows that in the MM based structure, LDOS peak associated to surface plasmon can be shifted to low energy direction, where absorption in metal reduces and therefore Purcell coefficient increases. As we show in the “Methods” section, the results obtained by effective media approach can be valid, if the thickness of the layers does not exceed dozens of nanometers. In realistic metamaterial, the thickness of the layers cannot be less than few nm for technological and fundamental reasons, so the range of layer thickness for realistic metamaterial, where the effect of reduction of surface plasmon frequency can be implemented is not large.

**Figure 5:**
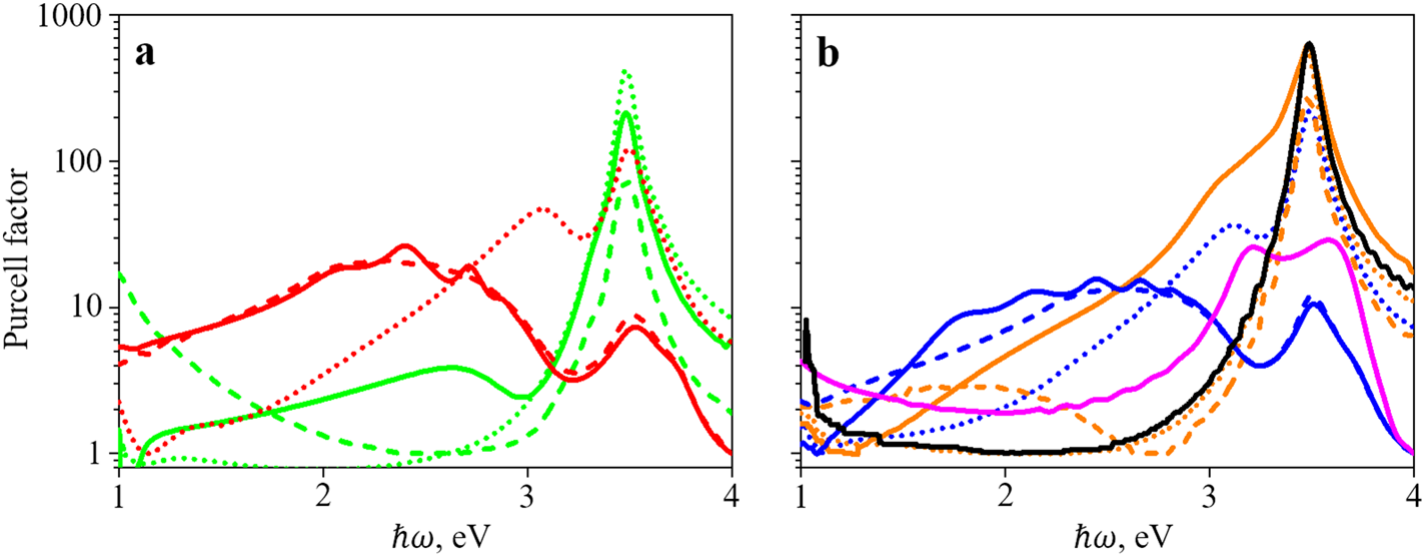
Dependence of the Purcell factor on frequency calculated via FDTD simulations for a silver/CBP metamaterial on silica substrate. Colors denote different values of $\alpha$ (red: $\alpha = 0.15$, blue: $\alpha = 0.2$, green: $\alpha = 0.7$, orange: $\alpha = 0.8$) in a silver/CBP metamaterial. Black and magenta lines denote Purcell factor of a simple plasmon for an interface between silver and CBP (magenta line) and silica (black line). Lines denote period length: solid line—0.5 nm; dashed line—5 nm; dotted line—15 nm. The dipole is parallel to the layers. The data was obtained using Lumerical R2 2020^41^ and plotted in OriginPro (ver. 8.6, https://www.originlab.com/). Images were combined using Microsoft PowerPoint 365 (https://www.office.com/).

Therefore, it is interesting to obtain spectrum of the Purcell coefficient obtained by numerical modeling of realistic structures based on metamaterial with various thicknesses of the layers. Figure 5 shows the spectrum of Purcell coefficients, obtained by the finite difference time-domain (FDTD) method for silver/CBP metamaterial with different period (15 nm, 5 nm, and 0.5 nm) and filling factors $\alpha = 0.15, \alpha=0.2,\alpha=0.7$ and $\alpha = 0.8$ on the silica substrate. The metamaterial consists of 21 pairs of layers of silver and CBP. The metamaterial with the period of the structure 0.5 nm can hardly be fabricated at present technological level, but it is useful to consider it as well for methodological reasons.

For comparison, the values of the Purcell factor for single interfaces silver/silica (dark yellow) and CBP/silica (yellow) are also shown. It can be seen, that for the silver silica interface the maximum of Purcell coefficient occurs for the photon energy 3.48 eV, and for the silver/CBP interface there are two maxima for the photon energy 3.2 eV and 3.6 eV, what coincides with the features shown in Fig. 1c.

For high filling factor $\alpha = 0.8$ the maximum value of the Purcell factor corresponds to the frequency of surface plasmons propagated along the silver/silica interfaces. While for lower filling factor, $\alpha = 0.2,$ an additional peak appears and decreasing the period length, and therefore approaching the effective medium approximation, the second peak is moving towards the lower frequencies, where the losses due to the absorption in metal (dispersive material) are smaller.

Comparison of Figs. 4a,b and 5 shows that the results of numerical modeling are qualitatively similar to the results obtained using effective media approach. It can be seen that additional features appear in the spectra of Purcell coefficient at the photon energies below the energies of surface plasmons at the interface silver/CBP and silver/silica. For the period of metamaterial 15 nm, there is small modification of the spectra for filing factor $\alpha = 0.8$, but for $\alpha = 0.2$ additional peak of the Purcell coefficient appears at the photon energy 3.1 eV. For the period of MM 5 nm, there is broadband increase of the Purcell coefficient in the interval of photon energies between 2 and 3 eV. Note that there are substantial discrepancies between positions and shape of the peaks of Purcell coefficient obtained by numerical modelling and effective media approach. For discussion on that we refer the reader to the Supplementary Discussion and Results, where also the results of numerical simulation for dipole perpendicular to the layers are presented.

Our results are in line with other results reported in works^32–38^, although the geometry and materials are different. It is hard to determine precisely the value of the Purcell factor in the previous studies^32,33,36^; however, judging from the decay time decrease, it should be in the range from 10 to 100. Similarly, the experiments from^34^ report the maximum value of 20, which is lower than we are predicting. This low value has to be attributed to the fact that the emitter in^34^ is made of quantum dots and, thus, is not distributed throughout the MM like in our case. It is also detached from the interface, which additionally leads to the decrease of the Purcell factor. Similarly, the numerical results in^37^ are obtained for a dipole, which is also detached. In^35^ the maximum value is around 100, which is consistent with our results. Finally, the review^38^ reports giant values of the Purcell factor around 1000, however, only for limit cases of $\varepsilon$, which makes it unrealizable and therefore of pure theoretical interest, while experimental values are below 100, repeating results of the previously mentioned works.

## Methods

### Analytic derivation for plasmon dispersion LDOS peak

Without loss of generality, we can assume that for all the waves in structure $k_{y} = 0$ due to the symmetry. The wavevector of the ordinary wave satisfies the following relation:

4 $$k_{x}^{2} + k_{z}^{2} = \varepsilon_{x} k_{0}^{2} ,$$

where $$k_{0} = \omega /c$$.

The wavevector of the extraordinary wave satisfies the equation:

5 $$\frac{{k_{x}^{2} }}{{\varepsilon_{z} }} + \frac{{k_{z}^{2} }}{{\varepsilon_{x} }} = k_{0}^{2}$$

If we denote the $z$-component of the wavevector in the dielectric medium as $k_{z1}$ and in the metamaterial as $k_{z2}$, then the boundary condition will give (apart from the conservation of the $k_{x}$ component) the following dispersion relation for an ordinary-type plasmon:

6 $$k_{x} = k_{0} \sqrt {\frac{{\varepsilon_{1} \varepsilon_{x} }}{{\varepsilon_{1} + \varepsilon_{x} }}}$$

This is similar to a conventional plasmon dispersion:

7 $$k_{x} = k_{0} \sqrt {\frac{{\varepsilon_{1} \varepsilon_{Me} }}{{\varepsilon_{1} + \varepsilon_{Me} }}}$$

For an extraordinary plasmon, the dispersion has the form:

8 $$k_{x} = k_{0} \sqrt {\frac{{\varepsilon_{1}^{2} \varepsilon_{z} - \varepsilon_{1} \varepsilon_{x} \varepsilon_{z} }}{{\varepsilon_{1}^{2} - \varepsilon_{x} \varepsilon_{z} }}}$$

We will show now that using a metamaterial can indeed shift the LDOS peak to the low-frequency range. If a metal is modelled by the Drude theory, its dielectric constant has the form

9 $$\varepsilon_{Me} \left( \omega \right) = \varepsilon_{0} - \frac{{\omega_{p}^{2} }}{{\omega \left( {\omega + i\gamma } \right)}}$$

For a metal–dielectric interface (when no metamaterial is used), the frequency for the LDOS peak can be estimated when the absorption ($\gamma$) is small:

10 $$\omega_{peak}^{\left( 0 \right)} = \frac{{\omega_{p} }}{{\sqrt {\varepsilon_{0} + \varepsilon_{1} } }}$$

Since the Drude theory can be used to describe the metal in the low-frequency range, we can apply several algebraic transformations to Eqs. (6) and (8) and get for an ordinary plasmon the following:

11 $$\omega_{peak}^{{\left( {ord} \right)}} = \frac{{\omega_{p} }}{{\sqrt {\varepsilon_{0} + \varepsilon_{1} + \frac{1 - \alpha }{\alpha }\left( {\varepsilon_{D} + \varepsilon_{1} } \right)} }}$$

And for an extraordinary plasmon, we obtain:

12a $$\omega_{peak}^{{\left( {ext} \right)}} = \frac{{\omega_{p} }}{{\sqrt {\varepsilon_{0} + \xi \pm \sqrt {\varepsilon_{1}^{2} + \xi^{2} } } }}$$

where $$\xi$$ reads

12b $$\xi = \frac{{\left( {1 - \alpha } \right)\left( {\varepsilon_{D}^{2} - \varepsilon_{1}^{2} } \right)}}{{2\alpha \varepsilon_{D} }}$$

It is clear that for an ordinary-wave plasmon, any value of $\alpha$ leads to a decrease in the peak frequency, while for an extraordinary one it is necessary to select “$+$. ” in “$\pm$” and satisfy $\xi > 0$ or $\varepsilon_{D} > \varepsilon_{1}$. Moreover, when $\xi = 0$ there is actually no difference in dispersion between the Eq. (8) and the usual Eq. (7), that is, using the same material for the fillingnd for the cladding will not lead to the appearance of a distinguishable extraordinary-wave plasmon.

Another important point is that

13 $$\mathop {\lim }\limits_{\alpha \to 0} \omega_{peak}^{{\left( {ord,ext} \right)}} = 0$$

For the extraordinary plasmon, this is valid only when $\varepsilon_{D} > \varepsilon_{1}$. In other words, by reducing the fraction of metal, we can achieve an arbitrarily small peak frequency—without changing the materials.

### Effective media method validity

Effective media approach is valid when the thickness of the layers forming the MM are much less than the inverse imaginary part of the lateral wavevector in the MM:

14 $$d \ll \kappa^{ - 1} , \kappa = Im\left( {k_{z2} } \right)$$

The value of $\kappa$ is derived from anisotropic Maxwell equations and has the following forms for the ordinary and extraordinary plasmons:

15 $$\kappa^{{\left( {ord} \right)}} = \sqrt {k_{x}^{2} - \varepsilon_{x} k_{0}^{2} } , \kappa^{{\left( {ext} \right)}} = \sqrt {\varepsilon_{x} \varepsilon_{z}^{ - 1} k_{x}^{2} - \varepsilon_{x} k_{0}^{2} }$$

For materials considered in this work the minimal value of $\kappa^{ - 1}$. just exceeds 100 nm.

### Purcell factor calculation

The values of the Purcell factor have been calculated using the method described in detail previously^21^. In brief, we solve the Maxwell equation by postulating that the wave propagates along the layers, and its amplitude vanishes when moving away from the interface. The resulting solution is then normalized and substituted into the Fermi golden rule when calculating dipole trantion matrix element. The dipole is placed on the interface and oriented along the layers, which corresponds to the maximum Purcell factor.

### FDTD calculations

FDTD calculations were done using Lumerical software package^41^. A perfectly-matched layer absorbing boundaries are applied at all edges of the simulation region to minimize reflections. For our simulations, the dipole source is oriented parallel to the layers and is located in the close proximity to the interface silica/silver, to meet the maximum of the electric field of the surface plasmon but maintaining some distance from the dispersive material. Additional details on meshing are to be found in Supplementary Material.

## Conclusion

To conclude, we have shown that the peak of local density states associated with the surface plasmon and the associated peak in the Purcell coefficient can be shifted towards lower energy, where the absorption of metal decreases if the bulk uniform metal is replaced by periodic metal-dielectric structures. The shift of the LDOS peak toward lower energy is accompanied by increase in the peak value of the Purcell coefficient. For the surface plasmon localized at the interface between the silver/CBP metamaterial and silica, the peak value of the Purcell coefficient can be shifted to visible/infrared energy range, where the value of the Purcell coefficient is increased by one order of magnitude. The results of numerical modeling of Purcell coefficient qualitatively support the results, obtained by effective media approach.

## Supplementary information

Supplementary information.
