# Supplementary material for: Control of the surface plasmon dispersion and Purcell effect at the metamaterial-dielectric interface
Source: Sci Rep. 2020 Nov 30;10:20828. doi: 10.1038/s41598-020-77688-6 (PMC7705705; doi:10.1038/s41598-020-77688-6)
Supplement: Supplementary file 1 — Supplementary information. [file 41598_2020_77688_MOESM1_ESM.docx]

## Supplementary information for the article

Control of the surface plasmon dispersion and Purcell effect at the metamaterial-dielectric interface

Konstantin A. Ivanov,^1,*^ Konstantin M. Morozov,^1,2^ Galia Pozina,^3^ Azat R. Gubaydullin,^1,2,4^ Elizaveta I. Girshova,^1,2^ and Mikhail A. Kaliteevski^1,2^

^1^ ITMO University, 197101 St. Petersburg, Russia

^2^ St. Petersburg Academic University, 194021 St. Petersburg, Russia

^3^ Department of Physics, Chemistry and Biology (IFM), Linköping University, SE-58183 Linköping, Sweden

^4^ Department of Applied Physics, Aalto University School of Science, P.O. Box 13500, 00076, Aalto, Finland

[**kivanov1992@gmail.com*](mailto:*kivanov1992@gmail.com)

# Extended results and discussion of the FDTD modelling

Results of numerical simulation presented in Fig. 5 depend on the FDTD setting particularly the mesh at the metal/dielectric interfaces is crucial. The mesh should be adjusted to materials, because the wavelength is smaller in the higher index material. Also, for the dipole calculations, there is a requirement of several mesh cells per metal layer to attain higher accuracy. Therefore, it is most accurate to compare FDTD results for a pair of the filling factors such as 0.2 and 0.8.

We perform FDTD simulations for several different periods (15 nm, 5 nm, and 0.5 nm), decreasing the period length to approach closer to the effective medium approximation (EMA) which is explained in detail in the “Methods” section of the main manuscript. We also perform the modal analysis and calculate the bandstructure, see Figure S1, which illustrates the complicated ensemble of eigenmodes in the considered system. Those surface plasmon modes are responsible for the complicated dependence of the Purcell factor presented in Figure 5 in the manuscript. We also vary the filling factor. For the maximum value of the filling factor $\alpha=1$ the conventional metal-dielectric interface plasmon is contributing the Purcell factor, see dark yellow in Figure 5. From a brief analysis, decreasing the filling factor simply reduces losses in the metal, as the metal portion in the period is decreasing, and enhances the confinement in the CBP layer. And surface plasmon propagating along the silver/CBP interfaces also contribute to the Purcell factor, see yellow in Figure 5. Figure 1 (b) shows that the imaginary part of the dielectric constant of CBP tends to zero for lower frequencies, which together with lower filling factor results in increase of the Purcell coefficient. The increase of Purcell coefficient is mainly affected by the contribution of the ordinary and extraordinary surface plasmon modes, which are best explained by the EMA. Unfortunately, it is complicated to investigate by FDTD independently the contribution of those modes in the metamaterial system, therefore, the analysis and the discussion in the main text are mainly focused on the EMA results. For very small values of the filling factor $\alpha=0$ there should be no surface plasmon at the dielectric/dielectric interface.

Comparing the results obtained by the numerical modeling and effective media approach substantial discrepancies between positions and shape of the peaks of the Purcell coefficient are observed. To distinguish the possible numerical artifacts and related unphysical modes we also perform the modal analysis and calculate the bandstructure, which is presented in Figure S1 (c) for the structure comprising 4 layers Ag 3 nm - gray, CBP 12 nm - light green, red –Si substrate, as shown in Figure S1 (a). The bandstructure analysis illustrates a sophisticated structure of multiple modes contributing to the Purcell factor calculated by FDTD presented on figure 5. Such a complicated band structure makes it difficult to distinguish separately the ordinary and extraordinary plasmon modes which we observe by our analytical model, hence the difference between Fig. 4 and Fig. 5. For comparison the bandstructure of a single Ag/CBP period on a Si substrate (as shown in Figure S1 (b)) is also simulated - Figure S1 (d).


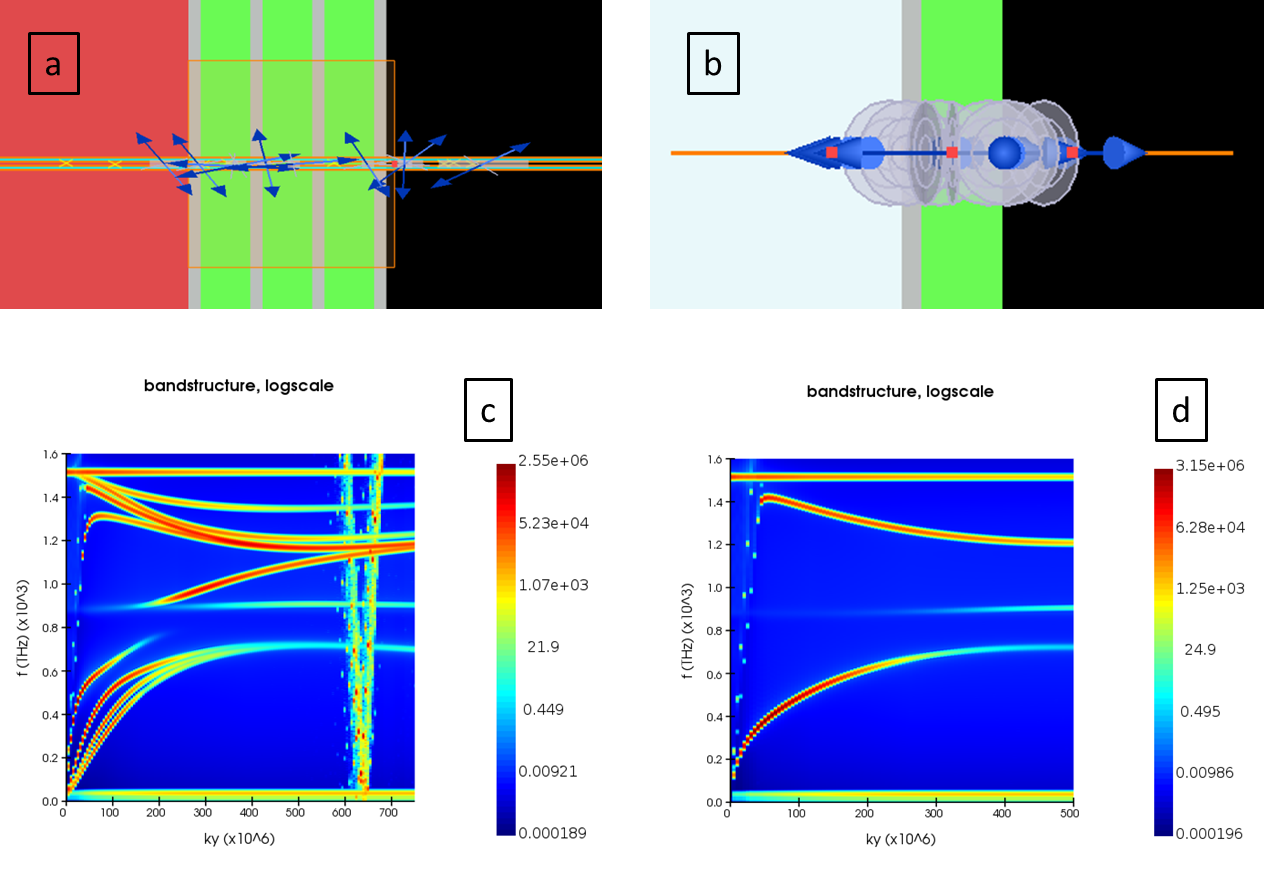


Figure S1. (a) The system for bandstructure analyses: 4 Ag layers 3 nm - gray, 3 CBP layers 12 nm - light green, red – Si substrate, and black considered as vacuum. (b) A system with single Ag/CBP period: Ag 3 nm - gray, CBP 12 nm - light green, cyan – Si substrate. (c) The bandstructure calculated for system (a). (d) The bandstructure calculated for system (b). The images and plots were created using Lumerical (ver. R2 2020, https://www.lumerical.com/products/).

Figure S2 presents the results of FDTD simulation when the dipole is perpendicular to the layers, which might be instructive for some purposes. As with effective media approximation, there’s little difference between orientations, particularly, Fig. S2 is very similar to Fig. 5a. We also note that we chose to estimate the Purcell factor in case when the dipole is parallel to layers because in this case the maximum of the dipole radiation could be considered of normal incidence to the interfaces and we consider the minimum possible periods, when differences related to effects of diffraction and the polarization dependence are less pronounced and almost negligible for the variation of the maximum value of the Q factor variation.


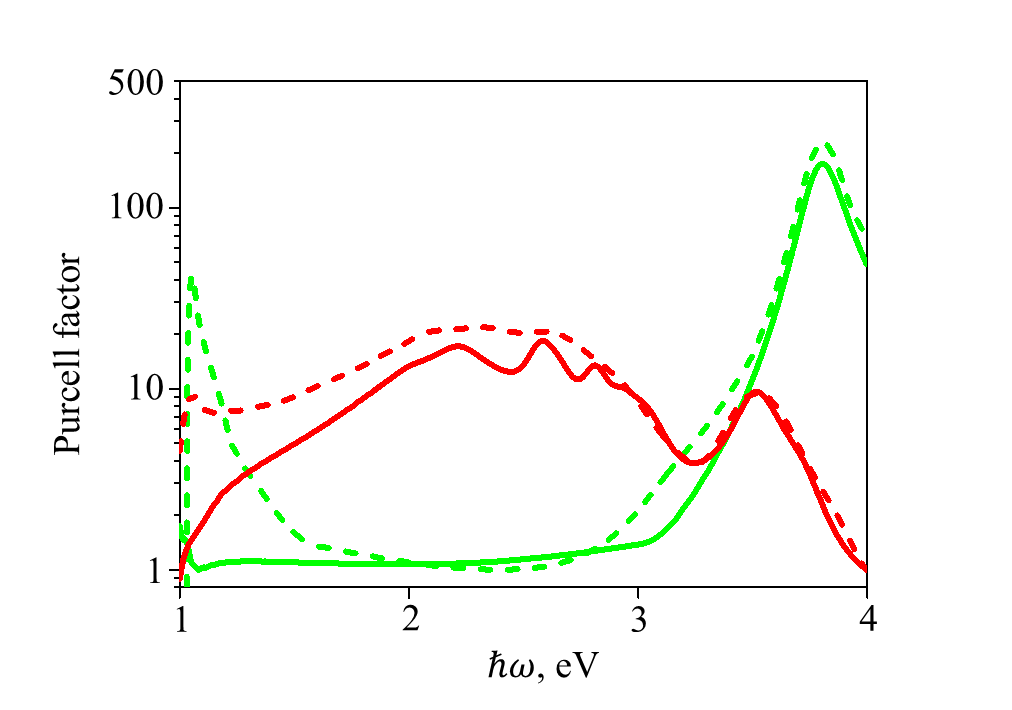


Figure S2. Dependence of the Purcell factor on frequency calculated via FDTD simulations for a silver/CBP metamaterial on silica substrate when the dipole is perpendicular to the interface. Two values of α (green: α=0.7, red: α=0.15) and different periods of the metamaterial are considered (solid: 0.5 nm, dashed: 5 nm). The data was obtained using Lumerical (ver. R2 2020, https://www.lumerical.com/products/) and plotted in OriginPro (ver. 8.6, https://www.originlab.com/).
